# Comment on “Maternal Satisfaction on Delivery Service and Its Associated Factors among Mothers Who Gave Birth in Public Health Facilities of Debre Markos Town, Northwest Ethiopia”

**DOI:** 10.1155/2015/320368

**Published:** 2015-09-06

**Authors:** Siddharudha Shivalli

**Affiliations:** Department of Community Medicine, Yenepoya Medical College, Yenepoya University, Mangalore, Karnataka 575018, India

I read the paper titled “Maternal Satisfaction on Delivery Service and Its Associated Factors among Mothers Who Gave Birth in Public Health Facilities of Debre Markos Town, Northwest Ethiopia” by Bitew et al. [1], with a great interest. Authors' efforts are highly commendable. It provides valuable information for evidence based interventions to enhance the quality and efficiency of maternal healthcare services in the study area. However, the following issues and concerns need to be addressed.

As it was a community based study and systematic random sampling was done, the following should have been mentioned: How many mothers were assessed for eligibility in the study area and how many of them were eligible and consented for voluntary participation (i.e., response rate)? These would enhance the internal validity of the findings.

Although authors mention “knowledge on ANC services” as one of the independent variables, but respective findings are not mentioned in the results.

It is not clear whether all or only the significant independent variables on univariate analysis were included in regression model. Authors should have mentioned the crude OR for all the independent variables considered in this study. And adequacy of the applied binary logistic regression model is not mentioned. Failure to do so may lead to misleading or incorrect inferences.

I feel that the following independent variables should have also been studied as they may influence the subjective feeling of satisfaction: predominant source of ANC (private or public health sector), gender of the baby, counseling, and level of birth preparedness and complication readiness.

Since satisfaction is very subjective (also quoted by the author) [2], top of mind response/s of the study participants as a feedback to improve the quality of healthcare services would have helped to fix the priorities.
